# Evolution of the M gene of the influenza A virus in different host species: large-scale sequence analysis

**DOI:** 10.1186/1743-422X-6-67

**Published:** 2009-05-29

**Authors:** Yuki Furuse, Akira Suzuki, Taro Kamigaki, Hitoshi Oshitani

**Affiliations:** 1 Department of Virology, Tohoku University Graduate School of Medicine, 2-1 Seiryou-machi Aoba-ku, Sendai, Japan

## Abstract

**Background:**

Influenza A virus infects not only humans, but also other species including avian and swine. If a novel influenza A subtype acquires the ability to spread between humans efficiently, it could cause the next pandemic. Therefore it is necessary to understand the evolutionary processes of influenza A viruses in various hosts in order to gain better knowledge about the emergence of pandemic virus. The virus has segmented RNA genome and 7th segment, M gene, encodes 2 proteins. M1 is a matrix protein and M2 is a membrane protein. The M gene may be involved in determining host tropism. Besides, novel vaccines targeting M1 or M2 protein to confer cross subtype protection have been under development. We conducted the present study to investigate the evolution of the M gene by analyzing its sequence in different species.

**Results:**

Phylogenetic tree revealed host-specific lineages and evolution rates were different among species. Selective pressure on M2 was stronger than that on M1. Selective pressure on M1 for human influenza was stronger than that for avian influenza, as well as M2. Site-by-site analyses identified one site (amino acid position 219) in M1 as positively selected in human. Positions 115 and 121 in M1, at which consensus amino acids were different between human and avian, were under negative selection in both hosts. As to M2, 10 sites were under positive selection in human. Seven sites locate in extracellular domain. That might be due to host's immune pressure. One site (position 27) positively selected in transmembrane domain is known to be associated with drug resistance. And, two sites (positions 57 and 89) locate in cytoplasmic domain. The sites are involved in several functions.

**Conclusion:**

The M gene of influenza A virus has evolved independently, under different selective pressure on M1 and M2 among different hosts. We found potentially important sites that may be related to host tropism and immune responses. These sites may be important for evolutional process in different hosts and host adaptation.

## Background

The influenza virus is a common cause of respiratory infection all over the world. The influenza A virus can infect not only humans but also avian, swine, and equine species. The virus has a negative single-stranded RNA with eight gene segments, namely PB2, PB1, PA, HA, NP, NA, M, and NS. The subtype of influenza A virus is determined by the antigenicity of two surface glycoproteins, hemaglutinin (HA) and neuraminidase (NA). The subtypes currently circulating in the human population are H1N1 and H3N2. Influenza A viruses cause epidemics and pandemics by antigenic drift and antigenic shift, respectively [1]. Antigenic drift is an accumulation of point mutations leading minor and gradual antigenic changes. Antigenic shift involves major antigenic changes by introduction of new HA and/or NA subtype into human population.

All known HA and NA subtypes are maintained in avian species, and all mammalian influenza A viruses are thought to be derived from the avian influenza A virus pool [1]. In avian species, influenza A viruses are in an evolutionary stasis [1]. In contrast, all gene segments of mammalian viruses continue to accumulate amino acid substitutions [1]. Today, the emergence of an influenza pandemic is of great global concern. If a novel influenza A subtype acquires the ability to spread between humans efficiently, it could cause the next pandemic [1]. This ability is acquired by reassortment between human and non-human influenza A viruses or by the accumulation of mutations in the non-human influenza virus. It is necessary to understand the evolutionary processes of influenza A viruses in various hosts so that we have better knowledge about the emergence of this pandemic virus. We conducted the present study to investigate the evolution of the M gene among different species. Although there are numerous studies on the evolution of the HA gene [2-7], only a few studies on the evolution of the M gene have been conducted [8].

The M gene is intriguing because it encodes both matrix and membrane proteins, and has multiple functions. The M gene (1027 bps) encodes two proteins, namely M1 (at nucleotide position 26 to 784) and M2 (at nucleotide position 26 to 51 and 740 to 1007) [9]. M1 is a matrix protein that lies just beneath the viral envelope in the form of dimers and interacts with viral ribonucleoprotein (vRNP) complex, forming a bridge between the inner core components and the membrane proteins [10-13]. vRNPs harbor the determinants for host range [1,14,15]. M1 contacts with both viral RNA and NP, promoting the formation of RNP complexes and causing the dissociation of RNP from the nuclear matrix [16-21]. M1 plays a vital role in assembly by recruiting the viral components to the site of assembly and essential role in the budding process including formation of viral particles [22,23]. M2 is a membrane protein which is inserted into the viral envelope and projects from the surface of the virus as tetramers [24,25]. The M2 protein comprises 97 amino acids – 24 in the extracellular domain, 19 in the transmembrane domain, and 54 in the cytoplasmic domain. Extracellular domain of M2 is recognized by hosts' immune system [26-28]. Transmembrane domain of M2 has ion channel activity, which involved in uncoating process of the virus in cell [29]. Amantadine inhibits virus replication by blocking the acid-activated ion channel. The cytoplasmic domain of M2 interacts with M1 and is required for genome packaging and formation of virus particles [30-36].

The molecular mechanism of how the host range of influenza A viruses is determined is still not fully understood. The M gene may be involved in determining host tropism. Besides, novel vaccines targeting M1 or M2 proteins to confer cross-subtype protection have been shown to be promising [37-43]. Therefore, understanding of evolution of the M gene is of great importance and practical relevance.

## Results

### Phylogenetic Tree

**Figure 1:**
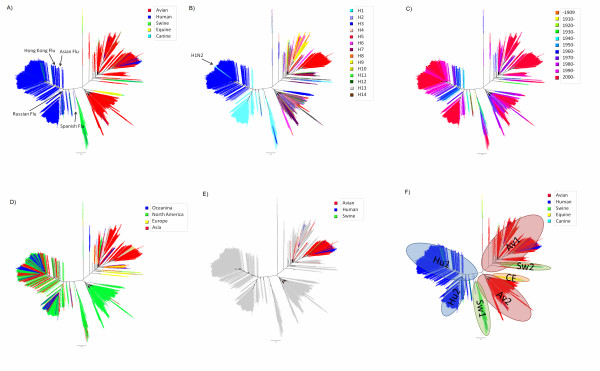
**Phylogenetic trees for the M gene**. Figures shows phylogenetic trees constructed using RAxML. Scale bar shows evolutionary distance inferred by RAxML algorithm. Trees are shaded in colors according to host (A), subtype (B), year (C), geographical location (D), and H5N1 (E). To compare evolutionary characteristics such as evolution rate and selective pressure, we named each lineage as shown in (F).

The phylogenetic trees for the M gene of all the sequence data we analyzed are shown in Figure 1. We defined "lineage" as an aggregate of large branches. The phylogenetic analysis revealed seven host-specific lineages: 1) human lineage (Hu1) consisting of H1N1 between 1918 and 1954 (Spanish Flu and its progeny viruses), H2N2 between 1957 and 1967 (Asian Flu and its progeny viruses), and H3N2 (Hong Kong Flu and its progeny viruses) after 1968; 2) another human lineage (Hu2) consisting of H1N1 (Russian Flu) after 1977; 3) avian lineage (Av1) including viruses mainly from Asia but also from other regions; 4) another avian lineage (Av 2) including viruses mostly from North America; 5) swine lineage (Sw1), located between human and avian lineages, mainly from North America; 6) another swine lineage (Sw2) diverging from Av1 and consisting of swine viruses after 1980, mainly from Europe; and 7) canine/equine lineage (CE) diverging from the root of Av2.

The M gene of all known human influenza A viruses, i.e., H1N1 between 1918 and 1957, H2N2 between 1957 and 1968, H3N2 after 1968, and H1N1 after 1977 was derived from that of the 1918 Spanish Flu. One lineage (Hu1) included three different subtypes (H1N1 between 1918 and 1957, H2N2 between 1957 and 1968, and H3N2 after 1968), which means that the same M gene was maintained in human influenza even after two antigenic shifts in 1957 and 1968. Another lineage (Hu2) included H1N1 after 1977. This M gene was also derived from Spanish Flu, but underwent different evolutionary processes and formed another lineage. Since H1N1 re-emerged in 1977 as Russian Flu, the two subtypes (H1N1 and H3N2) have been co-circulating in human populations and have formed two distinct lineages (Hu1 and Hu2). However, Hu2 exclusively includes H1N1 viruses and all human H3N2 are included in Hu1 (Figure 1B). On the other hand, both avian influenza lineages (Av1 and Av2) did not show any subtype specificity, and included many different subtypes (Figure 1A and 1B). In avian lineages, even small branches of the phylogenetic tree are shared by different subtypes.

Although strains with the M gene in both avian lineages (Av1 and Av2) have been seen sporadically in humans, they have not been maintained in the population (blue characters in Av1 and Av2, Figure 1A and 1F). Strains with the M gene in swine lineages also infect humans, but these swine viruses have not been established in human populations (blue characters in Sw1 and Sw2, Figure 1A and 1F). All H5N1 viruses that infected humans as well as the H5N1 virus that infected swine possessed share the M gene of the avian influenza lineage (Av1, (Figure 1E).

### Evolutionary Rate

**Figure 2:**
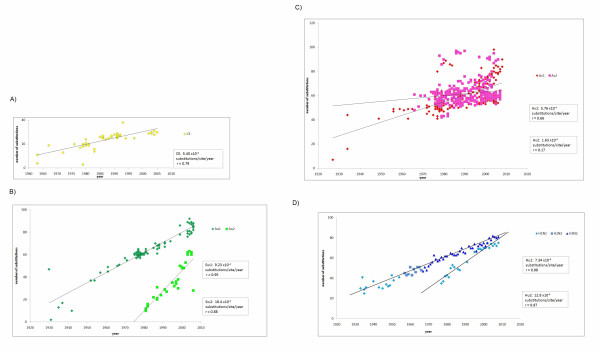
**Evolutionary rate**. Number of nucleotide substitutions compared to the oldest strain in each lineage is plotted. Evolutionary rates are calculated from the slope of the tangent of a simple regression line (number of substitutions/site/year), for canine/equine (A), swine (B), avian (C), and human (D). Correlation coefficient (r) was estimated using the Pearson correlation. Reference strains are A/chicken/Brescia/1902(H7N7) for Av1, A/turkey/Massachusetts/3740/1965(H6N2) for Av2, A/equine/Miami/1/1963(H3N8) for CE, A/Brevig Mission/1/1918(H1N1) for Hu1 and Hu2, A/swine/Iowa/15/1930(H1N1) for Sw1, and A/swine/Netherlands/25/80(H1N1) for Sw2. Mean and 95% confidence interval (shown in parentheses) are calculated by SPSS.

For evolutionary rate analysis, we included the sequences of only host-specific lineages and excluded other sequences such as those of the H5N1 influenza in humans (Figure 1F. See "Materials and methods"). The profile of the sequences analyzed is shown in Table 1. Evolutionary rates were estimated for each lineage (Figure 2).

**Table 1:** Profile of sequences analyzed for selective pressure

Host	Total number	Number after excluding identical sequences	Year	Mean diversity
All hosts	5060	3011	1902 – 2008	0.100
Human	2763	1217	1918 – 2008	0.050
Avian	2009	1492	1902 – 2008	0.077
Swine	201	123	1930 – 2006	0.069
Canine/Equine	87	53	1963 – 2005	0.015

Av2 of avian influenza A viruses showed the slowest evolutionary rate (1.63 × 10^-4 ^substitutions per site per year). All human and swine Influenza A viruses had a significantly faster evolutionary rate than avian viruses (Table 2). In addition, evolutionary rates were significantly different even between lineages of same host. Hu2 has evolved more rapidly than Hu1, and Sw2 has evolved more rapidly than Sw1 (Figure 2 and Table 2).

**Table 2:** Comparison of evolutionary rates among different hosts

		Av1	Av2	^a^within each host
	Evolutionary rate (number of substitutions/site/year)	5.76 × 10^-4^	1.63 × 10^-4^	
Hu1	7.34 × 10^-4^	**0.020**	**< 0.001**	
Hu2	12.8 × 10^-4^	**< 0.001**	**< 0.001**	**< 0.001**

Sw1	9.23 × 10^-4^	**< 0.001**	**< 0.001**	
Sw2	18.4 × 10^-4^	**< 0.001**	**< 0.001**	**< 0.001**

CE	5.40 × 10^-4^	0.795	**0.007**	

List of P-values for differential evolutionary rates.

^a^P-values for lineages of same host: Hu1 vs. Hu2 and Sw1 vs. Sw2.

Bold values are those deemed to show significantly positive selection (P < 0.05).

### Selective Pressures

**Figure 3:**
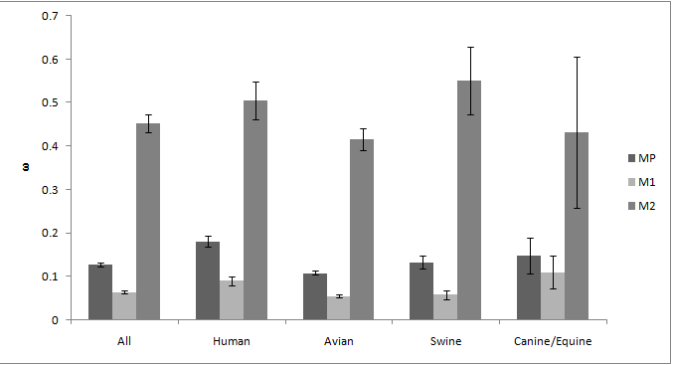
**Selective pressure among hosts**. Selective pressures for the entire sequence (ω) are calculated for the entire coding region of the M gene, and separately for M1 and M2. Error bar shows 95% confidence interval.

The selective pressures for the entire sequence (we defined the magnitude of the pressure as "ω") were 0.13 for the entire coding region of the M gene, 0.06 for M1, and 0.45 for M2 (Figure 3). A higher selective pressure indicates that the gene (or the site) is under stronger selection (positive selection) for amino acid substitution. Lower selective pressure indicates that the gene (or the site) is under stronger negative selection to retain the same amino acid(s) because changes may lead to incompetence or abortion [44,45]. Selective pressure was statistically stronger in M2 than that in M1 for all hosts.

ω of the entire coding region of the M gene for human and swine influenza was significantly higher (no overlap of 95% confidence intervals) than that for the avian influenza (Figure 3). ω for both M1 and M2 of human influenza are also significantly larger than that for avian influenza (Figure 3).

### Site-by-site Analyses

Site-by-site (by each codon) analyses for human influenza were conducted by SLAC (the entire tree [eSLAC], internal branches [iSLAC], and terminal branches [tSLAC]), and FEL (the entire tree [eFEL] and internal branches [iFEL]) methods [45]. We conducted the analyses by testing hypotheses for the entire tree, internal branches, and terminal branches (See "Materials and methods").

**Figure 4:**
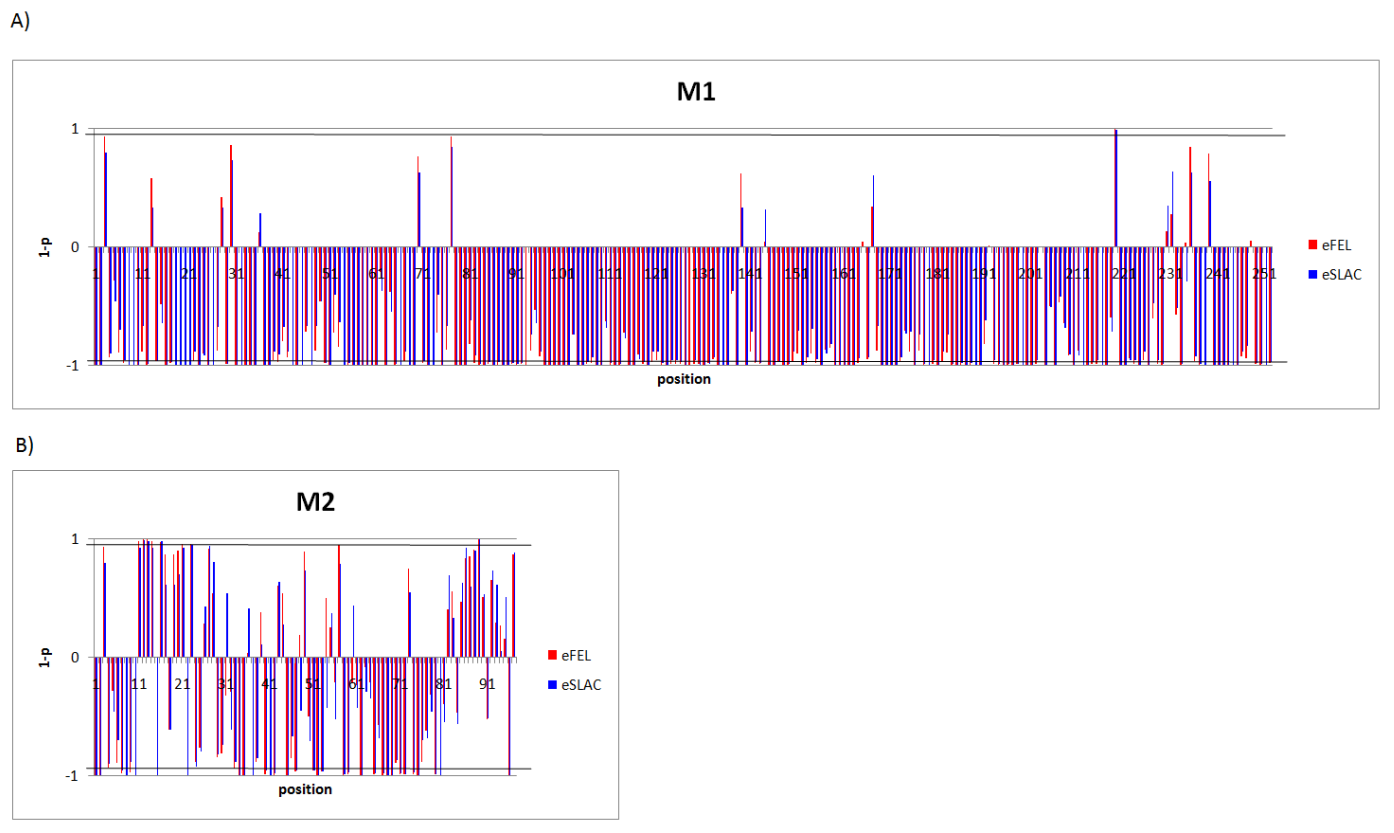
**Selection profile by eFEL and eSLAC**. Selection profiles of M1 (A) and M2 (B) are shown. The abscissa indicates the codon position. The ordinate indicates the (1-p) value for each position, and is above or below the horizontal line when dN/dS > 1 or dN/dS < 1, respectively. The horizontal lines represent 0.95, so that the positions where the bars cross the lines above and below indicate the positively and negatively selcected sites, respectively. The results of eSLAC and eFEL are shown.

**Figure 5:**
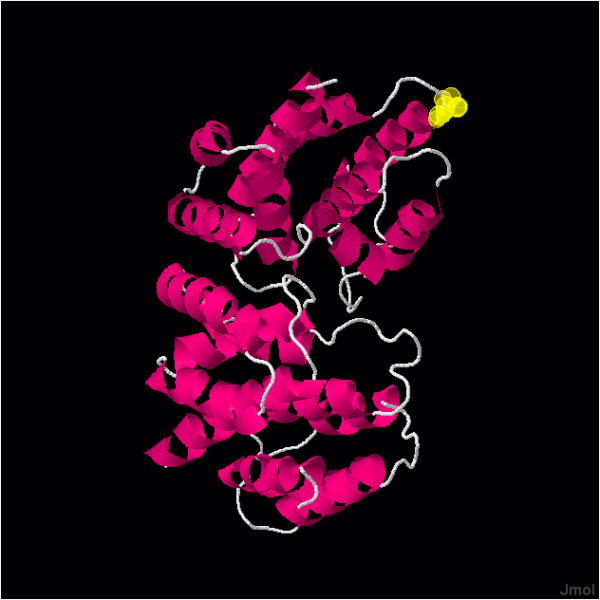
**3D crystral structure of M1**. The figure was generated using BioHealthBase. M1 is identified as dimers. Site at position 219 (yellow circles), which is under positive selection for human influenza, is located at the edge of the structure.

"dN/dS" indicates the magnitude of selective pressure on each codon. When dN/dS on a certain codon is significantly greater than 1, the site is considered to be under significant positive selection. When dN/dS on a certain codon is significantly smaller than 1, the site is considered to be under significant negative selection. Figure 4 shows P-values calculated by eSLAC and eFEL for each codon, indicating negative or positive selection. eSLAC and eFEL gave similar results. The sites under significant negative selection for human influenza were found in 159 out of 252 codons (63.1%) in M1 and 26 out of 97 (26.8%) in M2. Only one codon (0.4%) in M1 and eight codons (8.2%) in M2 were under significant positive selection by eFEL for human influenza. The sites under positive selection identified by at least one test are listed in Table 3. The site in M1 under significant positive selection was position 219 (from here, "position" indicates the amino acid position, i.e., the codon). Figure 5 shows that this site is located at the edge of the structure and is a part of a T-cell and MHC cell epitope. Of ten sites positively selected in M2, seven sites are in the extracellular domain (positions 11, 12, 13, 14, 16, 21, and 23), one site is in the transmembrane domain (position 27), and two sites in the cytoplasmic domain (positions 57 and 89, Table 3).

**Table 3:** Sites under positive selection for human influenza

Gene	Domain^a^	Position	dN/dS^b^	eSLAC	iSLAC	tSLAC	eFEL	iFEL
M1		219	inf^c^	**0.0048**	0.22	**0.022**	**0.0032**	**0.013**

M2	Ex	11	inf	0.074	0.23	0.33	**0.017**	**0.0055**
	Ex	12	inf	**0.0068**	0.32	**0.022**	**0.0026**	**0.024**
	Ex	13	inf	**0.020**	0.55	**0.036**	**0.0025**	0.064
	Ex	14	inf	0.069	0.37	0.19	**0.019**	**0.025**
	Ex	16	6.18	**0.015**	**0.027**	0.27	**0.021**	**0.0017**
	Ex	21	inf	0.071	0.37	0.19	**0.036**	**0.040**
	Ex	23	inf	0.052	0.27	0.19	**0.037**	**0.024**
	Tm	27	4.42	0.054	0.59	**0.039**	0.082	0.20
	Cy	57	3.62	0.21	0.16	0.57	0.050	**0.024**
	Cy	89	inf	**0.002**	0.12	**0.016**	**0.0023**	**0.0046**

The significance of SLAC and FEL results for positive selection levels are given as P-values.

^a^Ex indicates extracellular domain; Tr, transmembrane domain; and Cy, cytoplasmic domain.

^b^dN/dS was calculated by eFEL.

^c^"inf" means infinity as denominator is 0.

Bold values are those deemed to show significantly positive selection (P < 0.05).

**Figure 6:**
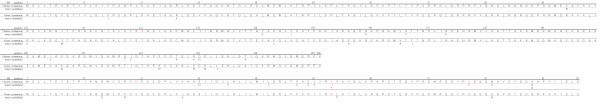
**Consensus sequence**. Consensus amino acid sequences of human and avian influenza A virus are shown. The major variable is defined as amino acid variants which are found in 10% or more strains. Different sites are shaded in red.

**Figure 7:**
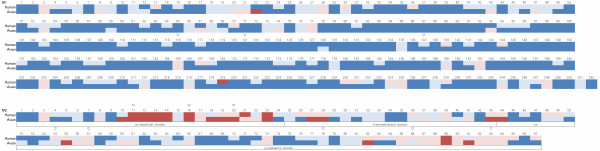
**Summary of site-by-site analyses**. The figure shows the positive or negative selection in human and avian influenza, and differences in consensus sequences between the hosts. Amino acid positions under positive and negative selection are shaded in red and blue, respectively. Sites under significant positive and negative selection are shaded in dark colors, while light colors indicate no significance. Triangles indicate sites where the consensus amino acids are different between human and avian influenza.

To define the evolutionary difference for each codon in human and avian influenza, we also calculated site-by-site selective pressures for avian influenza by eFEL. Consensus sequences of human and avian viruses were compared to identify major differences between these two hosts. We identified the sites at which consensus amino acids were different between the human and avian viruses and showed selective pressures (Figure 6 and Table 4). A summary of the site-by-site analyses including positive and negative selection for human and avian influenza, and differences in the consensus sequences are shown in Figure 7. Position 219 in M1, which is under significant positive selection in the human virus, is under significant negative selection in the avian virus. Positions 115 and 121 in M1, which are under significant negative selection in both human and avian viruses, have different consensus amino acids between the hosts (Figure 7 and Table 4).

**Table 4:** Selective pressure on different sites between human and avian influenza

			Human		Avian	
			
Gene	Domain^a^	Position	dN/dS^b^	P-value	dN/dS	P-value
M1		115	0.07	**0.0031**	0.06	**< 0.001**
		121	0.11	**0.039**	0.07	**< 0.001**
		137	0.69	0.61	0.03	**< 0.001**

M2	ex	11	inf^c^	**0.017**	inf	**< 0.001**
	ex	16	6.18	**0.021**	5.06	**0.044**
	ex	20	Inf	0.094	inf	**< 0.001**
	cy	54	1.36	0.50	0.50	0.12
	cy	57	3.62	0.050	inf	0.12
	cy	78	0.74	0.68	0.26	0.086
	cy	86	3.47	0.16	0.64	0.64
	cy	93	1.27	0.71	inf	0.067

^a^Ex indicates extracellular domain; Tr, transmembrane domain; and Cy, cytoplasmic domain.

^b^dN/dS was calculated by eFEL.

^c^"inf" means infinity as denominator is 0.

Significance of the FEL test for positive selection levels is given as P-values, and underlined values indicate P-values for negative selection.

Bold values are those deemed to indicate significantly positive or negative selection (P < 0.05).

## Discussion

The phylogenetic tree showed that the M gene of influenza A viruses has evolved independently in each host. It revealed host-specific lineages, which were compatible with other reports. In previous reports, Av1, Av2, Sw1, Sw2, and CE were named as Eurasian (Old World) avian, North American (New World) avian, classic (old) swine, European (avian-like) swine, and recent (avian-like) canine lineages, respectively [1,8,46,47]. Since the emergence of the Russian Flu, both H1N1 and H3N2 have been co-circulating in human populations and undergoing different evolutionary processes, which have resulted in two distinct human influenza lineages, Hu1 and Hu2 (Figure 1A, B, and 1F). Although reassortment of human influenza A viruses between the same subtype (intratypic recombination) has occurred frequently [48-51], we found only a few strains that seemed to be generated by reassortment between H1N1 and H3N2 human influenza, including H1N2 strains. These strains were not maintained in human populations. When the H3N2 virus with the M gene in Hu1 acquires the M gene from H1N1 in Hu2, such a virus might not replicate and/or transmit effectively. On the other hand, M genes of avian influenza are frequently shifted between subtypes as shown in Figure 1A and 1B. This suggests that reassortment between subtypes (intertypic recombination) is common in avian influenza. This result is compatible with the study by Dugan et al., which showed a high rate of gene reassortment among avian influenza A viruses [52]. It is still unclear why the M gene of avian influenza is interchangeable among subtypes, while the M gene of human influenza is not. Further experiments in vitro are necessary to answer this question.

After Spanish Flu, the same M gene has been maintained in human influenza, even after two pandemics (Asian Flu and Hong Kong Flu) that were thought to have been generated by reassortment between avian and human influenza A viruses [1] (Figure 1A and 1C). In the phylogenetic tree (Figure 1A), Spanish Flu is located at the root of a human lineage and close to a swine lineage; there is a greater distance between Spanish Flu and the avian influenza A viruses identified around 1918. This result supports the hypothesis that an ancestral virus of Spanish Flu had entered the mammalian population before 1918 [53,54]. It remains to be seen whether this M gene will be retained after further pandemics. It was shown that the M gene of recent human influenza cannot incorporate the HA segment of avian influenza in vitro [55].

There have been several sporadic infections with viruses from non-human lineages to humans, including the recent H5N1 infections in humans. However, these viruses were not maintained, and therefore, they disappeared from the human population without efficient transmission from human to human. In addition, it is implied that swine can be a "mixing vessel" in which human and avian viruses are reassorted to generate a human pandemic strain [1,56]. However, infections of strains with avian or human M genes in swine were also rare, and most of these viruses were not maintained in the swine population, except for the Sw2 lineage, in which viruses with the avian lineage M gene became established in the swine population.

Our phylogenetic analysis showed that viruses were clustered in host-specific lineages. This suggests that the M gene may be host specific and viruses with an M gene from other hosts are difficult to replicate. It is possible that the M gene determines the host range through the interaction between M1 and vRNPs [13,14,57]. An M gene that can match with host-specific vRNPs may be needed to replicate and transmit in a certain host. In addition, many studies have shown the interaction between M1 protein and host proteins, such as RACK1, MAPK, and core histone [13,58-60]. The M gene may be directly and/or indirectly linked to host tropism of the virus.

The evolutionary rate of the M gene was low in avian viruses compared to human and swine viruses (Figure 2 and Table 2). This result is rational because birds are considered to be a natural host for the influenza A virus [1]. The avian influenza A virus may have already been adapted to the host and not subject to pressure to induce further amino acid changes. This is also supported by the result showing that ω of the M gene was the lowest in avian influenza (Figure 3). Additional amino acid changes might be required in mammalian hosts to allow the viruses to adapt to these relatively new hosts. This stronger selective pressure on human and swine influenza may make human and swine influenza evolve more rapidly than avian influenza (Figures 2 and 3).

Interestingly, evolutionary rates were significantly different between lineages of the same host (Table 2). The evolutionary rates of Hu2 and Sw2 were faster than Hu1 and Sw1, respectively. The evolution of the M gene might not only be controlled by host species. One possible explanation is that strains in a lineage that appeared more recently such as Hu2 or Sw2, have to evolve more rapidly in order to be adapted better to the host than strains in other pre-existing lineages (Hu1 or Sw1), which have already adapted to some extent. Social factors at the time when new lineages appeared such as the growth of the population and globalization may also facilitate a faster evolution. This may be the reason why the evolutionary rates of Hu2 and Sw2 are higher than those of Hu1 and Sw1, respectively (Figure 2). However, reason of difference between evolutionary rates of Av1 and Av2 is unclear.

The selective pressure is stronger in M2 than in M1 (Figure 3) and more sites under positive selection were identified in M2 than in M1 (Table 3 and Figure 7). Among them, most of the sites (7 out of 10) under positive selection in M2 are located in the extracellular domain (Table 3 and Figure 7). Infection of influenza A virus induces the host's immune response to M2, especially to the extracellular domain [26-28]. It has been shown that antibodies recognizing the extracellular domain including the sites under positive selection confer protective immunity [37-39]. The host's immune response may make stronger selective pressure on M2 than that on M1. However, of course, selective pressure is much higher in the HA segment, the major antigenic component, than in the M2 gene [61], and this M2 gene is thus more conserved than the HA gene [42].

M1 is thought to play a vital role in the assembly and budding process [12,22,23]. Even minor mutations in M1 may cause a critical deficiency in virus replication. This could also explain why M1 is under strong negative pressure and why the selective pressure on M1 is smaller than that on M2 (Figure 3). Nevertheless, the selective pressure on M1 of the human influenza was stronger than that of the avian influenza (Figure 3). M1 of human influenza should be under stronger selective pressure than that of avian influenza to be better adapted.

Position 219 in M1 is under positive selection in human influenza. It was also reported that this site was positively selected using a different method of calculation [62]. However, this site is under negative selection in avian influenza (Figure 7). M1 is recognized by cytotoxic T cells [40,63,64] and the C-terminal of M1 determines antigenicity [65,66]. The site, located at the edge of structure (Figure 5), is part of the T-cell and MHC epitope. M1 may also be under selective pressure from the host's immune response, although this is weaker than M2. Besides, the C-terminal of M1 is important for binding to vRNPs [16]. This site might play an important role in the interaction with vRNPs, being associated with host range. Therefore, it is under positive selection only in the human and not in avian influenza virus.

Positions 115 and 121 in M1, which are under significant negative selection in both human and avian influenza, had different consensus amino acids between these two hosts (Figure 7). These results indicate that these sites may be important for host tropism and are therefore under negative selection. In addition, position 137 also has different consensus amino acids between the hosts, though this site is not under significant negative selection in human influenza (the site is under negative selection in avian influenza). The two domains in M1 have been reported to affect the disposition of viral RNA. One domain resides in a palindromic stretch of basic amino acids (position 101 to 105) [17,18] and the other domain is located at position 148 to 162 containing a zinc finger motif [19,20]. The three sites (positions 115, 121, and 137) are located between these two domains. These sites might affect the disposition of viral RNA and be involved in the determination of host range.

Position 27, which is a site in the transmembrane domain, is positively selected in M2. This site is associated with amantadine resistance [67]. The selective pressure on the site may be due to drug pressure. However, we could not show any positive pressure on position 31, which is associated with the recent spread of amantadine resistance [68]. Details on drug pressure and possible mechanism for recent surge of amantadine-resistant strains will be described in another manuscript (in preparation).

The cytoplasmic domain of M2 is important for interaction with M1, genome packaging, and formation of virus particles [33-36]. Two sites are under positive selection in the cytoplasmic domain of M2 (positions 57 and 89, Table 3). In particular, position 57 showed different consensus amino acids between human and avian influenza (Figure 7). These results indicate that the amino acids in these sites have frequently changed, and these sites are likely to be involved in several functions of M2. The M2 cytoplasmic tail (position 45 to 69) has been shown to be a binding domain for M1 [35]. Position 82 to 89 is important for infectious virus production [35]. Another study showed that vRNP packaging is mediated by amino acids at position 70 to 89 of the M2 gene [69]. The M2 gene must, therefore, have evolved with several functions.

In conclusion, the M gene of the influenza A virus has evolved with different selective pressures on M1 and M2 among different hosts. We found potentially important sites that may be related to host tropism and immune responses. These sites may be important for evolutionary processes in different hosts and host adaptation. However, Dunham et al. concluded that it is difficult to predict what specific genetic changes are needed for mammalian adaptation by comparing evolution of avian and swine influenza A viruses [47]. Further studies to clarify the specific role of each site identified in the present study are needed.

## Methods

### Sequence Data

All data were obtained from the influenza sequence database (Influenza Virus Resource on: , accessed on July 21, 2008) [70]. All sequencing data for the strains with a full-length M gene of any subtypes of influenza A from different host species including avian, canine, equine, human, and swine were included. Sequences derived from laboratory strains and duplicate strains verified by the strain name were excluded. A total of 5489 sequences were obtained [accession numbers are listed in additional file 1]. After excluding sequences containing ambiguous nucleotides, minor insertions, minor deletions (data for full length of coding region were used) or premature termination codons, a total of 5060 sequences were used in the analysis. Sequencing data were obtained together with information of the host, subtype, isolation year, and isolation place. The sequencing numbers for the influenza of each host are listed in Table 1. A multiple alignment of the nucleotide sequences, which did not contain any gaps, was constructed using ClustalW.

### Phylogenetic Tree Analysis

A phylogenetic tree was inferred by RAxML [71]. The sequences data only for the coding region were used; i.e., at nucleotide position 26 to 1007. The basic sequential algorithm of RAxML is described elsewhere [72]. RAxML is one of the fastest and most accurate sequential phylogeny programs [73]. In this method, a rapid bootstrap search is combined with a rapid maximum likelihood search on the original alignment. The tree was constructed using Web-servers, RAxML BlackBox: "" [71]. The tree is color-coded according to hosts, subtypes, geographical information, or temporal information using FigTree (ver.1.1.2).

### Dataset of Influenza for Each Host

Datasets for each host (avian, canine/equine, human, and swine hosts) were constructed. Sequences only from the host-specific lineage in the phylogenetic tree were used. For example, the H5N1 influenza A viruses that had infected humans were excluded from the analyses because humans were accidental hosts infected with the viruses of an avian lineage. Identical nucleotide sequences in the same dataset were removed before further analyses.

The number of base substitutions per site from an average of all sequence pairs was calculated to define the diversity of sequences in each dataset (Table 1) using the maximum composite likelihood method in MEGA (ver. 4) [74].

### Evolutionary Rate

The evolutionary rate of each lineage was calculated. To calculate the rate, at least one sequence of each subtype in each year was selected from each dataset. Evolutionary rate was analyzed for the selected sequences as the number of substitutions per site per year compared to the oldest strain in each lineage with a linear regression model. The significance of the correlations was estimated using the Pearson correlation. Differential between slopes of the tangent of simple regression lines were tested by analysis of covariance. The analyses were conducted using SPSS (ver.17).

### Consensus Sequence

Consensus amino acid sequences were determined as the sequence of amino acids that were identified most frequently at each position in a dataset, for human and avian influenza. Amino acid substitutions that were identified in more than 10% of the strains were regarded as major variants.

### Evaluation of Pressure (ω)

Phylogenetic trees for each dataset by hosts were constructed using the maximum-likelihood method implemented in PhyML-aLRT [75] with the GTR model (four rate categories, all parameters estimated from the data).

Selective pressure for each host population was calculated using the trees. Selective pressure was analyzed by HyPhy [76]. All analyses in HyPhy were conducted after identifying the best fit model from every possible time-reversible model (e.g., F81 and HKY85) according to Akaike's information criterion [45,77].

Global estimates (ω) of relative rates of non-synonymous (dN) and synonymous (dS) substitutions averaged over the entire alignment were compared to calculate the overall strength of selection [45].

### Site-by-site Selective Pressure (dN/dS)

Positive selection sites for human influenza were detected using two methods: single likelihood ancestor counting (SLAC) and fixed-effects likelihood (FEL). FEL was also conducted for avian influenza. The relative rates of non-synonymous and synonymous substitutions were compared. Sites where dN/dS > 1 and dN/dS < 1 were inferred as positively and negatively selected, respectively. The details of the two methods is described elsewhere [45,78,79]. It was shown that many recent non-synonymous substitutions, i.e., those in the terminal branches of the tree, were not represented on internal branches [80]. At codons where internal substitutions are seen, the strength of selection along the terminal branches is high. Analyses were conducted exclusively by testing hypotheses for the entire tree, internal branches, and terminal branches.

Briefly, in the SLAC method, the nucleotide and codon model parameter estimates are used to reconstruct the ancestral codon sequences at the internal nodes of the tree. The single most likely ancestral sequences are then fixed as known variables, and applied to infer the expected number of non-synonymous or synonymous substitutions that have occurred along each branch, for each codon position. SLAC is a substantially modified and improved derivative of the Suzuki-Gojobori method [44]. The FEL method is based on maximum-likelihood estimates. The FEL method estimates the ratio of non-synonymous to synonymous substitutions on a site-by-site basis for the entire tree (eFEL) or only the interior branches (iFEL). iFEL is essentially the same as eFEL, except that selection is only tested along the internal branches of the phylogeny [80].

Uniprot ("") and BioHealthBase ("") were used to generate 3D crystal structures and to determine the location of epitope sites. Accession numbers: [GeneBank protein GI 89779323, strain A/Puerto Rico/8/34, PDB ID 1AA7, GeneBank sequence accession CY009445].

In the present study, we used a newly developed softwares RAxML [71] and HyPhy [76] for phylogenetic analyses. Markov Chain Monte Carlo (MCMC) and PhyML are widely used and are considered useful for manipulating data sets (hundreds) [2,81-84]. However, they cannot process huge data sets in order of thousands on an ordinary desktop computer. Therefore, we used a PC with Windows operating system and 3 GB RAM). PAML, which is a common software package for phylogenetic analyses such as the calculation of selective pressure using maximum likelihood [85], also failed occasionaly in analyzing large data sets. We therefore used the recently developed software, which overcomes these problems. The accuracy of this software has been confirmed [73,79,86]. Through site-by-site analyses, we identified more sites negatively or positively selected than those in a study by Suzuki [61]. This was ascribed to a difference in the number of data sets and/or algorithms, as we analyzed ten times more than the number analyzed in his study.

## List of abbreviations

vRNP: viral ribonucleoprotein.

## Competing interests

The authors declare that they have no competing interests.

## Authors' contributions

YF carried out all analyses and drafted the manuscript. AS, TK, and HO participated in the design of the study and helped to draft the manuscript. All authors have read and approved the final manuscript.

## Supplementary Material

Additional file 1 **List of accession numbers**. The file contains list of accession numbers of sequencing data we analyzed. Click here for file

## References

[B1] Webster RG, Bean WJ, Gorman OT, Chambers TM, Kawaoka Y (1992). Evolution and ecology of influenza A viruses. Microbiological Reviews.

[B2] Zhang W, Jiang Q, Chen Y (2007). Evolution and variation of the H3 gene of influenza A virus and interaction among hosts. Intervirology.

[B3] Bush RM, Fitch WM, Bender CA, Cox NJ (1999). Positive selection on the H3 hemagglutinin gene of human influenza virus A. Mol Biol Evol.

[B4] Fouchier RAM, Munster V, Wallensten A, Bestebroer TM, Herfst S, Smith D, Rimmelzwaan GF, Olsen B, Osterhaus ADME (2005). Characterization of a novel influenza A virus hemagglutinin subtype (H16) obtained from black-headed gulls. Journal of Virology.

[B5] Bean WJ, Schell M, Katz J, Kawaoka Y, Naeve C, Gorman O, Webster RG (1992). Evolution of the H3 influenza virus hemagglutinin from human and nonhuman hosts. Journal of Virology.

[B6] Fitch WM, Leiter JM, Li XQ, Palese P (1991). Positive Darwinian evolution in human influenza A viruses. Proceedings of the National Academy of Sciences of the United States of America.

[B7] Shih AC-C, Hsiao T-C, Ho M-S, Li W-H (2007). Simultaneous amino acid substitutions at antigenic sites drive influenza A hemagglutinin evolution. Proceedings of the National Academy of Sciences of the United States of America.

[B8] Ito T, Gorman OT, Kawaoka Y, Bean WJ, Webster RG (1991). Evolutionary analysis of the influenza A virus M gene with comparison of the M1 and M2 proteins. Journal of Virology.

[B9] Lamb RA, Lai CJ, Choppin PW (1981). Sequences of mRNAs derived from genome RNA segment 7 of influenza virus: colinear and interrupted mRNAs code for overlapping proteins. Proceedings of the National Academy of Sciences of the United States of America.

[B10] Nayak DP, Hui EK-W, Barman S (2004). Assembly and budding of influenza virus. Virus Research.

[B11] Schmitt AP, Lamb RA (2005). Influenza virus assembly and budding at the viral budozone. Advances in Virus Research.

[B12] Cros JF, Palese P (2003). Trafficking of viral genomic RNA into and out of the nucleus: influenza, Thogoto and Borna disease viruses. Virus Research.

[B13] Garcia-Robles I, Akarsu H, Muller CW, Ruigrok RWH, Baudin F (2005). Interaction of influenza virus proteins with nucleosomes. Virology.

[B14] Naffakh N, Tomoiu A, Rameix-Welti M-A, Werf S van der (2008). Host restriction of avian influenza viruses at the level of the ribonucleoproteins. Annual Review of Microbiology.

[B15] Almond JW (1977). A single gene determines the host range of influenza virus. Nature.

[B16] Baudin F, Petit I, Weissenhorn W, Ruigrok RW (2001). In vitro dissection of the membrane and RNP binding activities of influenza virus M1 protein. Virology.

[B17] Elster C, Larsen K, Gagnon J, Ruigrok RW, Baudin F (1997). Influenza virus M1 protein binds to RNA through its nuclear localization signal. Journal of General Virology.

[B18] Wakefield L, Brownlee GG (1989). RNA-binding properties of influenza A virus matrix protein M1. Nucleic Acids Research.

[B19] Elster C, Fourest E, Baudin F, Larsen K, Cusack S, Ruigrok RW (1994). A small percentage of influenza virus M1 protein contains zinc but zinc does not influence in vitro M1-RNA interaction. Journal of General Virology.

[B20] Nasser EH, Judd AK, Sanchez A, Anastasiou D, Bucher DJ (1996). Antiviral activity of influenza virus M1 zinc finger peptides. Journal of Virology.

[B21] Ye Z, Liu T, Offringa DP, McInnis J, Levandowski RA (1999). Association of influenza virus matrix protein with ribonucleoproteins. Journal of Virology.

[B22] Gomez-Puertas P, Albo C, Perez-Pastrana E, Vivo A, Portela A (2000). Influenza virus matrix protein is the major driving force in virus budding. Journal of Virology.

[B23] Latham T, Galarza JM (2001). Formation of wild-type and chimeric influenza virus-like particles following simultaneous expression of only four structural proteins. Journal of Virology.

[B24] Lamb RA, Zebedee SL, Richardson CD (1985). Influenza virus M2 protein is an integral membrane protein expressed on the infected-cell surface. Cell.

[B25] Holsinger LJ, Lamb RA (1991). Influenza virus M2 integral membrane protein is a homotetramer stabilized by formation of disulfide bonds. Virology.

[B26] Gerhard W, Mozdzanowska K, Furchner M, Washko G, Maiese K (1997). Role of the B-cell response in recovery of mice from primary influenza virus infection. Immunological Reviews.

[B27] Potter CW, Oxford JS (1979). Determinants of immunity to influenza infection in man. British Medical Bulletin.

[B28] Treanor JJ, Tierney EL, Zebedee SL, Lamb RA, Murphy BR (1990). Passively transferred monoclonal antibody to the M2 protein inhibits influenza A virus replication in mice. Journal of Virology.

[B29] Pinto LH, Holsinger LJ, Lamb RA (1992). Influenza virus M2 protein has ion channel activity. Cell.

[B30] Zebedee SL, Lamb RA (1989). Growth restriction of influenza A virus by M2 protein antibody is genetically linked to the M1 protein. Proceedings of the National Academy of Sciences of the United States of America.

[B31] Hughey PG, Roberts PC, Holsinger LJ, Zebedee SL, Lamb RA, Compans RW (1995). Effects of antibody to the influenza A virus M2 protein on M2 surface expression and virus assembly. Virology.

[B32] Schroeder C, Heider H, Moncke-Buchner E, Lin T-I (2005). The influenza virus ion channel and maturation cofactor M2 is a cholesterol-binding protein. European Biophysics Journal.

[B33] Chen BJ, Leser GP, Jackson D, Lamb RA (2008). The influenza virus M2 protein cytoplasmic tail interacts with the m1 protein and influences virus assembly at the site of virus budding. Journal of Virology.

[B34] Castrucci MR, Kawaoka Y (1995). Reverse genetics system for generation of an influenza A virus mutant containing a deletion of the carboxyl-terminal residue of M2 protein. Journal of Virology.

[B35] McCown MF, Pekosz A (2006). Distinct domains of the influenza a virus M2 protein cytoplasmic tail mediate binding to the M1 protein and facilitate infectious virus production. Journal of Virology.

[B36] Ozawa M, Maeda J, Iwatsuki-Horimoto K, Watanabe S, Goto H, Horimoto T, Kawaoka Y (2009). Nucleotide sequence requirements at the 5' end of the influenza A virus M RNA segment for efficient virus replication. Journal of Virology.

[B37] Zharikova D, Mozdzanowska K, Feng J, Zhang M, Gerhard W (2005). Influenza type A virus escape mutants emerge in vivo in the presence of antibodies to the ectodomain of matrix protein 2. Journal of Virology.

[B38] Liu W, Zou P, Chen Y-H (2004). Monoclonal antibodies recognizing EVETPIRN epitope of influenza A virus M2 protein could protect mice from lethal influenza A virus challenge. Immunology Letters.

[B39] Zebedee SL, Lamb RA (1988). Influenza A virus M2 protein: monoclonal antibody restriction of virus growth and detection of M2 in virions. Journal of Virology.

[B40] Lee LY, Ha DL, Simmons C, de Jong MD, Chau NV, Schumacher R, Peng YC, McMichael AJ, Farrar JJ, Smith GL (2008). Memory T cells established by seasonal human influenza A infection cross-react with avian influenza A (H5N1) in healthy individuals[see comment]. Journal of Clinical Investigation.

[B41] Zhirnov OP, Isaeva EI, Konakova TE, Thoidis G, Piskareva LM, Akopova II, Kartashov A, Altstein AD, Ilyinskii PO, Shneider AM (2007). Protection against mouse and avian influenza A strains via vaccination with a combination of conserved proteins NP, M1 and NS1. Influenza Other Respir Viruses.

[B42] Fiers W, De Filette M, Birkett A, Neirynck S, Min Jou W (2004). A "universal" human influenza A vaccine. Virus Research.

[B43] Roose K, Fiers W, Saelens X (2009). Pandemic preparedness: Toward a universal influenza vaccine. Drug News & Perspectives.

[B44] Suzuki Y, Gojobori T (1999). A method for detecting positive selection at single amino acid sites. Mol Biol Evol.

[B45] Sergei L, Kosakovsky Pond AFYP, Simon DW (2007). Frost: Estimating selection pressures on alignments of coding sequences Analyses using HyPhy.

[B46] Reid AH, Taubenberger JK, Fanning TG (2004). Evidence of an absence: the genetic origins of the 1918 pandemic influenza virus. Nature Reviews Microbiology.

[B47] Dunham EJ, Dugan VG, Kaser EK, Perkins SE, Brown IH, Holmes EC, Taubenberger JK (2009). Different evolutionary trajectories of European avian-like and classical swine H1N1 influenza A viruses. Journal of Virology.

[B48] Furuse Y, Suzuki A, Kamigaki T, Shimizu M, Fuji N, Oshitani H (2009). Reversion of Influenza A (H3N2) from Amantadine-resistant to Amantadine-sensitive by Further Reassortment in Japan during the 2006–2007 Influenza Season. J Clin Microbiol.

[B49] Simonsen L, Viboud C, Grenfell BT, Dushoff J, Jennings L, Smit M, Macken C, Hata M, Gog J, Miller MA, Holmes EC (2007). The genesis and spread of reassortment human influenza A/H3N2 viruses conferring adamantane resistance. Molecular Biology & Evolution.

[B50] Holmes EC, Ghedin E, Miller N, Taylor J, Bao Y, St George K, Grenfell BT, Salzberg SL, Fraser CM, Lipman DJ, Taubenberger JK (2005). Whole-genome analysis of human influenza A virus reveals multiple persistent lineages and reassortment among recent H3N2 viruses. Plos Biology.

[B51] Nelson MI, Simonsen L, Viboud C, Miller MA, Taylor J, George KS, Griesemer SB, Ghedin E, Sengamalay NA, Spiro DJ, Volkov I, Grenfell BT, Lipman DJ, Taubenberger JK, Holmes EC (2006). Stochastic processes are key determinants of short-term evolution in influenza a virus. PLoS Pathog.

[B52] Dugan VG, Chen R, Spiro DJ, Sengamalay N, Zaborsky J, Ghedin E, Nolting J, Swayne DE, Runstadler JA, Happ GM (2008). The evolutionary genetics and emergence of avian influenza viruses in wild birds. PLoS Pathogens.

[B53] Reid AH, Fanning TG, Hultin JV, Taubenberger JK (1999). Origin and evolution of the 1918 "Spanish" influenza virus hemagglutinin gene[see comment]. Proceedings of the National Academy of Sciences of the United States of America.

[B54] Taubenberger JK, Reid AH, Krafft AE, Bijwaard KE, Fanning TG (1997). Initial genetic characterization of the 1918 "Spanish" influenza virus[see comment]. Science.

[B55] Scholtissek C, Stech J, Krauss S, Webster RG (2002). Cooperation between the hemagglutinin of avian viruses and the matrix protein of human influenza A viruses. Journal of Virology.

[B56] Scholtissek C, Burger H, Kistner O, Shortridge KF (1985). The nucleoprotein as a possible major factor in determining host specificity of influenza H3N2 viruses. Virology.

[B57] Huang X, Liu T, Muller J, Levandowski RA, Ye Z (2001). Effect of influenza virus matrix protein and viral RNA on ribonucleoprotein formation and nuclear export. Virology.

[B58] Takizawa N, Watanabe K, Nouno K, Kobayashi N, Nagata K (2006). Association of functional influenza viral proteins and RNAs with nuclear chromatin and sub-chromatin structure. Microbes & Infection.

[B59] Reinhardt J, Wolff T (2000). The influenza A virus M1 protein interacts with the cellular receptor of activated C kinase (RACK) 1 and can be phosphorylated by protein kinase C. Veterinary Microbiology.

[B60] Pleschka S, Wolff T, Ehrhardt C, Hobom G, Planz O, Rapp UR, Ludwig S (2001). Influenza virus propagation is impaired by inhibition of the Raf/MEK/ERK signalling cascade. Nature Cell Biology.

[B61] Suzuki Y (2006). Natural selection on the influenza virus genome. Molecular Biology & Evolution.

[B62] Bragstad K, Nielsen LP, Fomsgaard A (2008). The evolution of human influenza A viruses from 1999 to 2006: a complete genome study. Virology Journal.

[B63] Gotch F, McMichael A, Smith G, Moss B (1987). Identification of viral molecules recognized by influenza-specific human cytotoxic T lymphocytes. Journal of Experimental Medicine.

[B64] Jameson J, Cruz J, Ennis FA (1998). Human cytotoxic T-lymphocyte repertoire to influenza A viruses. Journal of Virology.

[B65] Ye ZP, Pal R, Fox JW, Wagner RR (1987). Functional and antigenic domains of the matrix (M1) protein of influenza A virus. Journal of Virology.

[B66] Bucher D, Popple S, Baer M, Mikhail A, Gong YF, Whitaker C, Paoletti E, Judd A (1989). M protein (M1) of influenza virus: antigenic analysis and intracellular localization with monoclonal antibodies. Journal of Virology.

[B67] Hay AJ, Wolstenholme AJ, Skehel JJ, Smith MH (1985). The molecular basis of the specific anti-influenza action of amantadine. EMBO Journal.

[B68] Bright RA, Medina M-j, Xu X, Perez-Oronoz G, Wallis TR, Davis XM, Povinelli L, Cox NJ, Klimov AI (2005). Incidence of adamantane resistance among influenza A (H3N2) viruses isolated worldwide from 1994 to 2005: a cause for concern[see comment]. Lancet.

[B69] McCown MF, Pekosz A (2005). The influenza A virus M2 cytoplasmic tail is required for infectious virus production and efficient genome packaging. Journal of Virology.

[B70] Bao Y, Bolotov P, Dernovoy D, Kiryutin B, Zaslavsky L, Tatusova T, Ostell J, Lipman D (2008). The influenza virus resource at the National Center for Biotechnology Information. Journal of Virology.

[B71] Stamatakis A, Hoover P, Rougemont J (2008). A rapid bootstrap algorithm for the RAxML Web servers. Systematic Biology.

[B72] Felsenstein J (1981). Evolutionary trees from DNA sequences: a maximum likelihood approach. Journal of Molecular Evolution.

[B73] Computing Large Phylogenies with Statistical Methods: Problems & Solutions. http://icwww.epfl.ch/~stamatak/index-Dateien/publications/BGRS2004.PDF.

[B74] Tamura K, Nei M, Kumar S (2004). Prospects for inferring very large phylogenies by using the neighbor-joining method. Proceedings of the National Academy of Sciences of the United States of America.

[B75] Anisimova M, Gascuel O (2006). Approximate likelihood-ratio test for branches: A fast, accurate, and powerful alternative. Syst Biol.

[B76] Pond SLK, Frost SDW, Muse SV (2005). HyPhy: hypothesis testing using phylogenies. Bioinformatics.

[B77] Lanave C, Preparata G, Saccone C, Serio G (1984). A new method for calculating evolutionary substitution rates. Journal of Molecular Evolution.

[B78] Campo DS, Dimitrova Z, Mitchell RJ, Lara J, Khudyakov Y (2008). Coordinated evolution of the hepatitis C virus. Proceedings of the National Academy of Sciences of the United States of America.

[B79] Kosakovsky Pond SL, Frost SD (2005). Not so different after all: a comparison of methods for detecting amino acid sites under selection. Mol Biol Evol.

[B80] Pond SLK, Frost SDW, Grossman Z, Gravenor MB, Richman DD, Brown AJL (2006). Adaptation to different human populations by HIV-1 revealed by codon-based analyses. PLoS Computational Biology.

[B81] Russell CA, Jones TC, Barr IG, Cox NJ, Garten RJ, Gregory V, Gust ID, Hampson AW, Hay AJ, Hurt AC, de Jong JC, Kelso A, Klimov AI, Kageyama T, Komadina N, Lapedes AS, Lin YP, Mosterin A, Obuchi M, Odagiri T, Osterhaus AD, Rimmelzwaan GF, Shaw MW, Skepner E, Stohr K, Tashiro M, Fouchier RA, Smith DJ (2008). The global circulation of seasonal influenza A (H3N2) viruses. Science.

[B82] Yang Z, Rannala B (1997). Bayesian phylogenetic inference using DNA sequences: a Markov Chain Monte Carlo Method. Mol Biol Evol.

[B83] Guindon S, Gascuel O (2003). A simple, fast, and accurate algorithm to estimate large phylogenies by maximum likelihood. Systematic Biology.

[B84] Barry G, Hall E (2007). Phylogenetic Trees Made Easy: A How-To Manual.

[B85] Yang Z (1997). PAML: a program package for phylogenetic analysis by maximum likelihood. Computer Applications in the Biosciences.

[B86] Llopart A, Comeron JM (2008). Recurrent events of positive selection in independent Drosophila lineages at the spermatogenesis gene roughex. Genetics.

